# Thermoneutrality-Induced Macrophage Accumulation in Brown Adipose Tissue Does Not Impair the Tissue’s Competence for Cold-Induced Thermogenic Recruitment

**DOI:** 10.3389/fendo.2020.568682

**Published:** 2020-10-30

**Authors:** Alexander W. Fischer, Jasper M. A. de Jong, Frederike Sass, Christian Schlein, Joerg Heeren, Natasa Petrovic

**Affiliations:** ^1^ Department of Molecular Biosciences, The Wenner-Gren Institute, Stockholm University, Stockholm, Sweden; ^2^ Department of Biochemistry and Molecular Cell Biology, University Medical Center Hamburg-Eppendorf, Hamburg, Germany

**Keywords:** brown fat, thermogenic capacity, UCP1, macrophages, thermoneutrality

## Abstract

Brown adipose tissue from mice living under conditions approaching human thermal and nutritional conditions (prolonged exposure to thermoneutral temperature and to an energy-rich (high-fat, high-sugar) diet) — referred to as “physiologically humanized” mice, displays morphological and molecular characteristics significantly different from those observed in young, chow-fed mice maintained at room temperature — referred to as “standard” mice. Here, we further examined brown fat from physiologically humanized and standard mice, as well as from mice exposed to thermoneutrality for a long time but not to an energy-rich diet - referred to here as “long-term thermoneutral” mice. Global transcriptome analysis of brown fat revealed that genes that were the most upregulated in brown fat of thermoneutral mice (both physiologically humanized and long-term thermoneutral) were those related to inflammatory processes, including genes expressed selectively in macrophages. Cellular and molecular analyses confirmed that brown fat from thermoneutral mice was heavily infiltrated by macrophages, predominantly organized into crown-like structures. However, despite this, the brown fat of thermoneutral mice retained full competence to attain the greatest possible recruitment state and became macrophage-depleted during the process of cold acclimation. Thus, profound macrophage accumulation does not influence the thermogenic recruitment competence of brown fat.

## Introduction

Brown fat is a highly heterogeneous tissue characterized by extraordinary plasticity. In response to changing environmental and metabolic conditions, brown adipose tissue undergoes a considerable and purposeful remodeling and attains a different recruitment state that fulfills the new thermogenic demands (1). Importantly, not only mature brown adipocytes, but also the other cell types (e.g. endothelial cells, preadipocytes, nerve terminals), go through qualitative and/or quantitative modifications in a coordinated manner which together ensure the requisite thermogenic activity of the tissue. We recently demonstrated that brown adipose tissue from mice exposed to conditions approaching human thermal and nutritional conditions - prolonged exposure to thermoneutral temperature (approximately 30°C) and to an energy-rich (high-fat, high-sugar) diet - undergoes remarkable morphological, cellular and molecular remodeling (2). In the physiologically humanized mice, brown fat was composed predominantly of unilocular brown adipocytes with only few UCP1-positive multilocular brown adipocytes. Notably, the tissue displayed a distinct molecular signature: in comparison to brown fat of young mice housed under standard conditions (temperature of approximately 21°C and chow diet), the expression levels of genes related to thermogenesis were significantly decreased, the expression levels of genes proposed to discriminate between classical brown and brite/beige adipose depots (marker genes) (3–6) were altered to such a degree that their brown versus brite/beige discriminative power was principally lost and as many as about one quarter of all transcripts were expressed at significantly different levels.

In the current study, we further analyze differentially expressed genes. We find that the greatest proportion of genes upregulated in brown fat upon “physiological humanization” are genes related to inflammatory pathways, including genes selectively expressed in macrophages. We concordantly observe a massive, thermoneutrality-dependent macrophage accumulation in the tissue. As adipose tissue inflammation has been widely (but not universally) suggested to be one of the major mechanisms underlying adipose tissue dysfunction, such an inflammatory process could be anticipated to permanently destroy or diminish the ability of the tissue to regain thermogenic competence. However, we find that the occurrence of these abundant macrophages does not result in detrimental effects on subsequent cold-induced recruitment of the tissue. Long-term thermoneutral conditions thus do not impede the attainment of the recruited state of brown adipose tissue.

## Materials and Methods

### Animals

All experiments were approved by the Animal Ethics Committee of the North Stockholm region. The experiments were performed on C57BL/6 male mice exposed to a 12:12-h light-dark cycle with free access to water and chow diet (Labfor R70; Lantmännen, Södertälje, Sweden) unless otherwise stated.

#### Standard Conditions (Young Mice)

The samples analyzed in this study were from mice described and analyzed in (2). C57BL/6 male mice were from Charles River and were kept in their original cages at 21°C until 8 weeks of age.

#### Standard Conditions (Middle-Aged Mice)

C57BL/6 male mice bred at the institute and remained in their original cages at 21°C until 11 months of age.

#### Physiologically Humanized Conditions

The samples analyzed in this study were from the wild-type mice (C57BL/6) described and metabolically characterized in (7) and molecularly and morphologically analyzed in (2). Before the start of the experiment, the mice were housed at 22°C to 24°C. At the start of the experiment, 12-week-old male mice were single-caged and transferred to 30°C (thermoneutrality). The mice had access only to high-fat diet (45% calories from fat, Research Diets D12451). The mice were kept at thermoneutrality for at least 25 weeks.

#### Long-Term Thermoneutrality

The samples analyzed in this study were from wild-type mice (C57BL/6) described and metabolically characterized in (7). At the start of the experiment, 12-week-old male mice were single caged and transferred to 30°C (thermoneutrality). The mice were kept at thermoneutrality for at least 25 weeks.

#### Time Course of Exposure to Thermoneutrality

C57BL/6 male mice bred at the institute remained at 21°C until 8 weeks of age. Then, the mice were exposed to 30°C (thermoneutrality) for 3 or for 7 days, or remained at room temperature until 9 weeks of age. Mice exposed to thermoneutrality for 30 days were from a different cohort (therefore indicated with stippled line, **Figure 4**); C57BL/6 male mice bred at the institute remained at 21°C until 11, 14 or 15 weeks of age and then were exposed to 30°C for 30 days.

#### Short-Term Thermoneutrality and Conventional Cold Acclimation

Samples in this study were obtained from male C57BL/6N mice purchased from the Charles River Laboratories and described in (8). At the start of the experiment, 8-week-old C57BL/6N male mice were single-caged and acclimated to thermoneutrality (30°C) or, in parallel, successively acclimated to cold by first placing them at 18°C for 1 week and then at 4°C for the following 4 to 5 weeks.

#### Cold Acclimation Of Long-Term Thermoneutral Mice

The samples analyzed in this study were from mice described and analyzed in (2). C57BL/6 male mice were purchased from Charles River. At 12 weeks of age, the mice (4 mice per cage) were transferred to 30°C (thermoneutrality). After 26 weeks at thermoneutrality, half of these long-term thermoneutral mice were sacrificed. Another half of these long-term thermoneutral mice were single-caged and successively acclimated to cold by first placing them at 18°C for 1 week and then at 4°C for the following 6 weeks.

#### Cold Acclimation of Standard 8 Months Old Mice

The samples analyzed in this study were from mice described and analyzed in (2). C57BL/6 male mice, bred at the institute, remained in their original cages at 21°C until 8 months of age. Then, the mice were single-caged and half of these mice remained at 21°C, while the other half were successively acclimated to cold by first placing them at 18°C for 1 week and then at 4°C for the following 6 weeks.

### Sampling of Tissues

At the end of the experiments, mice were euthanized with CO_2_. Interscapular brown adipose tissue (IBAT) was quantitatively dissected. The left and right lobes were placed in separate tubes and were either directly frozen in liquid nitrogen and stored at −80°C or fixed in alcoholic formaldehyde solution for histological analysis. The analysis of samples from mice exposed to different experimental conditions was performed in parallel, as indicated in Results/Figure legends.

### RNA Analysis

Total RNA was extracted from frozen IBAT (left lobe) with TRI Reagent^®^ (Sigma-Aldrich) according to the manufacturer’s protocol, and RNA concentrations were measured on a Nanodrop nd-1000 spectrophotometer (Thermo-Scientific, Wilmington, DE).

RNA sequencing was performed as described in (2).

Principal component analysis (PCA) was performed in MATLAB v.9.4.0.813654 using the pca function. The expression levels of macrophage marker genes analyzed in **Figure 4M** were calculated directly from RNA sequencing data. In each sample, the number of reads of the gene of interest was divided by the number of reads for TFIIB and multiplied by the ratio of TFIIB transcript length and the length of the transcript of the gene of interest (in bp). The expression levels of thermogenesis-related genes analyzed in **Figure 7H** were obtained by quantitative real-time PCR. The scores for PC1 and PC2 were plotted. The 67% confidence intervals are presented.

Differential gene expression analysis was performed using the DESeq2 algorithm on the RNA-Seq 2G web portal (http://52.90.192.24:3838/rnaseq2g/) on raw read counts. Significantly differentially expressed genes were defined based on fold change ≥ 2 and FDR (false discovery rates) ≤ 5%.

Venn diagram was created using Venn Diagram Plotter (https://omics.pnl.gov/software/venn-diagram-plotter; Pacific Northwest National Laboratory). Significantly differentially expressed genes were defined based on fold change ≥ 2 and adjusted P values < 0.05.

Consensus pathway analysis using ConsensusPathDB-mouse (http://cpdb.molgen.mpg.de/MCPDB; Max Planck Institute for Molecular Genetics) was performed to identify biological processes over-represented in the genes commonly upregulated in IBAT of thermoneutral versus standard mice.

Quantitative real-time PCR: To synthesize cDNA, 500 ng RNA was reverse-transcribed with a High Capacity cDNA kit (Applied Biosystems, Foster City, CA) in a total volume of 20 μl. Primers (**Supplementary Table 1**) were pre-mixed with SYBR^®^ Green JumpStart™ Taq ReadyMix™ (Sigma-Aldrich), and aliquots of 11 μl were applied to 96-well Multiplate^®^ PCR Plates™ (Bio-Rad). cDNA was diluted 1:10, and aliquots of 2 μl were added in triplicates. Thermal cycling conditions were: 2 min at 50°C, 10 min at 95°C, and 40 cycles of 15 s at 95°C and 1 min at 60°C on a CFX Connect™ Real-Time System (Bio-Rad). The ΔC_t_ method was used to calculate relative changes in mRNA abundance. The C_t_ value for transcription factor IIB (TFIIB), or for 18S rRNA, was subtracted from the C_t_ value for the target gene to adjust for variations in the efficiency of the cDNA synthesis. The values thus represent the number of RNA molecules per TFIIB mRNA molecule, or per 18S rRNA molecule (**Supplementary Figure 1**).

Hierarchical cluster analysis was performed on the ClustVis web tool (https://biit.cs.ut.ee/clustvis/) using Euclidean distance and average linkage based on expression values obtained with quantitative real-time PCR.

### Protein Analysis

Western Blot Analysis. The right lobe of IBAT was homogenized in modified RIPA buffer (50 mM Tris·HCl, pH 7.4, 1% Triton X-100, 150 mM NaCl, 1 mM EDTA) with freshly added 1 mM Na_3_VO_4_, 10 mM NaF and protease inhibitor cocktail (Complete-Mini, Roche). The homogenates, after freezing (in liquid nitrogen) and defrosting — in order to fully lyse brown adipocytes — were centrifuged at 14,000*g* for 15 min. The top fat layer was discarded and the lysate (infranatant) carefully aspirated using a 1-ml syringe and 27 G needle. The concentration of proteins in the lysate was determined using the Lowry method.

An equal volume of reducing sample buffer (125 mM Tris·HCl, pH 6.8, 4% (wt/vol) SDS, 20% (vol/vol) glycerol, 100 mM dithiothreitol, and 0.1% (wt/vol) bromphenol blue) was added to each sample. Proteins were separated by SDS-PAGE in ordinary 12% polyacrylamide gel (acrylamide/bis-acrylamide = 37.5/1). Proteins were transferred to polyvinylidene difluoride membranes (BioRad) in 48 mM Tris·HCl, 39 mM glycine, 0.037 (wt/vol) SDS and 15% (vol/vol) methanol, using a semi-dry electrophoretic transfer cell (Bio-Rad Trans-Blot SD; Bio-Rad) at 1.2 mA/cm^2^ for 90 min. After washing, the membrane was blocked in 5% milk in Tris-buffered Saline-Tween for 1 h at room temperature and probed with the indicated antibodies overnight at 4°C. The immunoblot was visualized with appropriate horseradish peroxidase-conjugated secondary antibodies and enhanced chemiluminescence (ECL kit, GE Healthcare Life Sciences) in a charge-coupled device camera (Fuji Film).

Antibodies used were as follows: UCP1 antibody (rabbit polyclonal, raised against C-terminal decapeptide), diluted 1:15000; MAC-2 antibody (Santa Cruz Laboratories, sc-23938), diluted 1:5000; perilipin antibody (Cell Signaling, #9349), diluted 1:2000; tyrosine hydroxylase antibody (Abcam, ab137869), diluted 1:5000.

Immunohistochemistry. One lobe of IBAT was immersion-fixed in 4% alcoholic formaldehyde (4% formaldehyde in ethanol) for 24 h, then dehydrated and embedded in paraffin by a standard procedure (9). Tissues were sectioned using a standard microtome (Leica RM2255, Leica Microsystems). Sections, 5 μm thick, were mounted on SuperFrost^®^ Plus adhesion slides (VWR International bvba, Leuven, Belgium), then deparaffinized and rehydrated. To unmask antigenicity, deparaffinized and rehydrated slides were boiled in citrate buffer (10 mM sodium citrate, pH 6) in a water bath for 30 min and cooled on the bench top for 30 min. Sections were then incubated in 0.3% Sudan Black B (Sigma-Aldrich, 199664) in 70% ethanol for 30 min at room temperature to block auto-fluorescence. Slides were rinsed with PBS and then placed in a humid chamber for incubation with blocking solution (3% BSA in PBS) for 2 h at room temperature. Negative controls were run to detect auto-fluorescence and any nonspecific binding. Primary antibodies were diluted in 1% BSA in PBS and a volume of 50-100 μl was pipetted on each tissue section for 24 h incubation at 4°C in a humid chamber. Antibodies used were as follows: MAC-2 (Santa Cruz Laboratories, sc-23938), diluted 1:100; perilipin (Cell Signaling, #9349), diluted 1:500 and tyrosine hydroxylase (Abcam, ab137869), diluted 1:100. In tissue sections immunostained for UCP1 (diluted 1:500), perilipin was visualized using an antibody produced in goat (Abcam, ab61682) diluted 1:250. After primary antibody incubation, the slides were washed with PBS for 1 h and then incubated with secondary antibodies diluted in 1% BSA in PBS; a volume of 50-100 μl was pipetted on each tissue section for 2 h incubation at room temperature in a humid chamber. Secondary antibody dilutions were 1:200 for chicken anti-rat Alexa Fluor 488-labeled secondary antibody (Molecular Probes, A21470), 1:500 for goat anti-rabbit Alexa Fluor 594-labeled secondary antibody (Molecular Probes, A11037), 1:500 for chicken anti-rabbit Alexa Fluor 488-labeled secondary antibody (Molecular Probes, A21441) and 1:500 for donkey anti-goat Alexa Fluor 594-labeled secondary antibody (Molecular probes, A11058). After secondary antibody incubation, sections were washed with PBS for 1 h. To stain nuclei, the sections were incubated in 1 μg/ml Hoechst 33258 (Sigma-Aldrich, 861405) for 10 min, washed with PBS for 30 min and mounted with ProGold^®^ antifade reagent (Molecular Probes, P36934). Slides were kept in the dark after secondary antibody incubation. Sections were analyzed in a confocal Zeiss LSM 780 microscope (Carl Zeiss Micro Imaging).

### Data Processing and Statistics

Data analysis was performed in Microsoft Excel 2016 and GraphPad Prism 8. Results are reported as the mean ± S.E. For statistical analysis, Student’s t-test or one-way ANOVA with a Tukey post-test were used, as indicated in the figure legends; P ≤ 0.05 was considered to be statistically significant. Multiple linear regression analysis was performed in GraphPad Prism 8. The dependent variable was gene expression and independent variables were temperature and age. For temperature, thermoneutrality was set as 1 and room temperature as 0. For age, middle age was set as 1 and young age as 0.

## Results

### Induction of Inflammatory Genes in Brown Fat Upon Prolonged Exposure to Thermoneutrality

**Figure 1:**
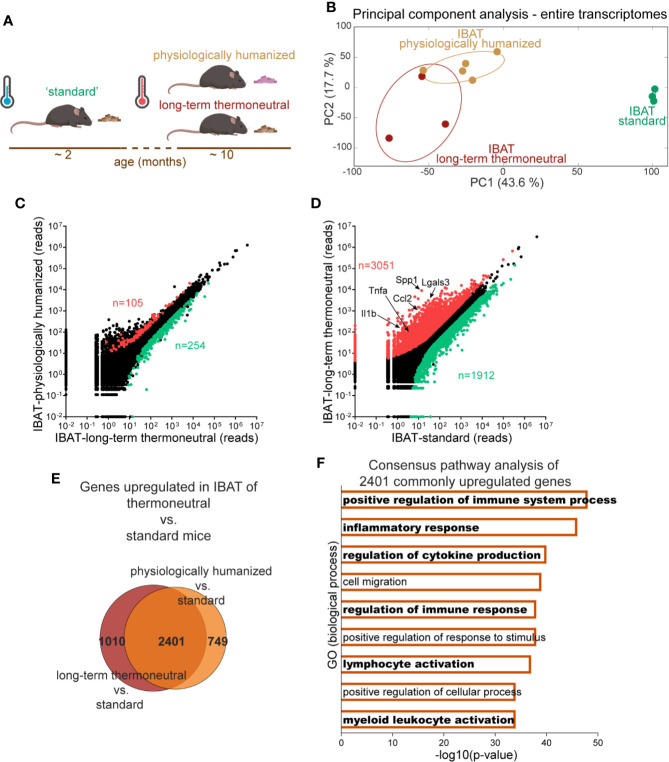
Induction of inflammatory genes in brown fat upon prolonged exposure to thermoneutrality. The transcriptomes of IBAT from physiologically humanized mice (n = 5), long-term thermoneutral mice (n = 3) and mice exposed to standard conditions (n = 3) were analyzed by RNA sequencing. **(A)** Schematic representation of experimental groups. **(B)** Principal component (PC) analysis of whole transcriptomes in the indicated samples (the samples were analyzed with RNA Sequencing). Each symbol represents one sample (mouse). Numbers in parentheses on the axes represent the proportion of data variance explained by each principal component. **(C)** Scatter plot presenting mean read counts for each gene in IBAT of long-term thermoneutral mice (x-axis) versus IBAT of physiologically humanized mice (y-axis). Genes significantly changed (fold change ≥ 2, FDR ≤ 5%) are colored in red and green for upregulated and downregulated, respectively. **(D)** Scatter plot presenting mean read counts for each gene in IBAT of mice exposed to standard conditions (x-axis) versus IBAT of long-term thermoneutral mice (y-axis). Genes significantly changed (fold change ≥ 2, FDR ≤ 5%) are colored in red and green for upregulated and downregulated, respectively. Example genes are indicated. **(E)** Venn diagram depicting genes upregulated in IBAT of physiologically humanized or long-term thermoneutral versus mice exposed to standard conditions (fold change ≥ 2, adjusted P values < 0.05). **(F)** Consensus pathway analysis of the 2401 genes upregulated in IBAT of both physiologically humanized and long-term thermoneutral mice versus mice exposed to standard conditions. Pathways related to inflammation are highlighted in bold.

Gene expression in brown fat is mainly determined by the environmental conditions that the animal is (or was) exposed to (1). “Standard” experimental mice are young, chow-fed and maintained at room temperature (approximately 21°C), which is much lower than the thermoneutral temperature for mice (approximately 30°C). Therefore standard mice are constantly exposed to cold stress. Such standard mice may be compared to “physiologically humanized” mice: middle-aged mice living under conditions approaching human thermal and nutritional conditions (prolonged exposure to thermoneutral temperature and to an energy-rich (high-fat, high-sugar) diet) (schematically depicted in **Figure 1A**). The transcriptome profiles of brown fat from standard and physiologically humanized mice exhibit significant differences (2), as is also seen in the principal component analysis (PCA) in **Figure 1B**.

The physiologically humanized mice are not only acclimated to a higher environmental temperature but also to a diet different from that of the standard mice. To identify the genes associated with this difference in diet we also included in the analysis the transcriptome of “long-term thermoneutral” mice (in parallel exposed to thermoneutrality but not to an energy-rich diet) (**Figure 1A**). IBAT samples from long-term thermoneutral and physiologically humanized mice assembled closely in PCA plot, which indicates a high degree of similarity of their transcriptomes (**Figure 1B**). In contrast, IBAT samples from standard mice formed a separate cluster positioned far from the other samples. In line with this, the number of genes that were differentially expressed between brown fat of long-term thermoneutral and physiologically humanized mice was relatively low - less than 2% (**Figure 1C**). In contrast, between the brown fat of standard and long-term thermoneutral mice, the expression levels of some 25% of the genes were different, even though both groups were fed a chow diet (**Figure 1D**). Thus, both analyses indicated that the effect of the environmental temperature on transcriptome changes by far exceeded the effect of the diet.

To identify biological processes associated with this prominent transcriptome remodeling, consensus pathway analysis was performed on genes that were commonly upregulated (2401 genes, **Figure 1E**) or downregulated in brown fat of both groups of thermoneutral mice versus mice exposed to standard conditions. Gene ontology analysis of the most downregulated genes revealed that biological processes associated with cellular respiration, fatty acid oxidation and mitochondrial activity were diminished upon prolonged exposure to thermoneutrality (not shown). However, notably, analysis of the most upregulated genes uncovered pronounced immune and inflammatory responses (**Figure 1F**, **Supplementary Figure 2** and **Supplementary Dataset 1**).

### Macrophage Gene Expression in Brown Fat

The above analysis suggested that the number and/or activity of immune cells present in the tissue increases upon prolonged exposure to thermoneutrality. A large proportion of the genes that were most upregulated upon prolonged exposure to thermoneutrality were genes that are selectively expressed in macrophages (e.g. *Spp1, Lgals3*) (highlighted in **Figure 1D**). As macrophages are the most prominent type of immune cells in adipose tissues - in terms of their number and function (10, 11) - the focus of this study was specifically to examine the occurrence of this type of immune cells in brown fat.

**Figure 2:**
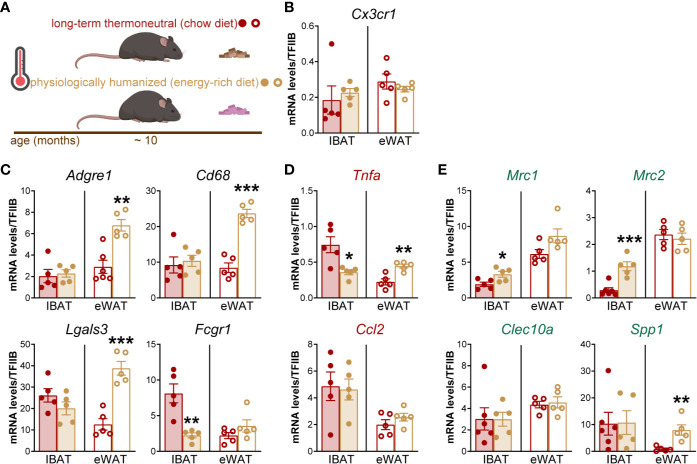
The effect of an energy-rich diet on thermoneutrality-driven macrophage gene expression in IBAT and eWAT. **(A)** Schematic representation of experimental groups. **(B**–**E)** Gene expression levels of tissue-resident macrophage marker gene **(B)**, general macrophage marker genes **(C),** M1 macrophage marker genes **(D)** and M2 macrophage marker genes **(E)** in IBAT and eWAT of long-term thermoneutral (n = 5) and physiologically humanized mice (n = 5). The tissues were analyzed in parallel. Values are means ± S.E. *P < 0.05; **P < 0.01; ***P < 0.001, significant difference between diets by Student’s unpaired t-test. The significance of difference between IBAT and eWAT was not calculated because the expression of TFIIB in IBAT and eWAT was different (**Supplementary Figure 1**).

To validate the RNA sequencing results, we measured the expression of macrophage marker genes in IBAT of long-term thermoneutral and physiologically humanized mice (schematically depicted in **Figure 2A**). A set of genes that are predominantly and/or selectively expressed in macrophages compared to brown adipocytes were selected for the analysis. Marker gene of tissue-resident macrophages (**Figure 2B**), general macrophage marker genes (**Figure 2C**), as well as both pro-inflammatory (M1 subtype) (**Figure 2D**) and anti-inflammatory (M2 subtype) (**Figure 2E**), macrophage marker genes were readily detected in IBAT of both groups of thermoneutral mice. The expression levels of macrophage marker genes in BAT were largely unaffected by diet and, in general, were of the same order of magnitude as in epididymal WAT of long-term thermoneutral mice. Thus, exposure to an energy-rich diet under thermoneutral conditions did not influence macrophage accumulation in brown fat (either proliferation of tissue-resident macrophages or macrophage infiltration into brown fat). We therefore subsequently concentrated on one of the thermoneutrality models: the long-term thermoneutrality with chow diet.

The absence of effect of diet is markedly different from the situation in visceral (epididymal) WAT, which both at standard temperature (12–14) and at thermoneutrality (**Figure 2** and (7)), demonstrates much higher expression of general and pro-inflammatory macrophage marker genes in mice on an energy-rich diet as compared to mice on chow diet.

### Macrophages That Accumulate in Brown Fat Upon Prolonged Exposure to Thermoneutrality Are Mostly Aggregated Into Crown-like Structures

**Figure 3:**
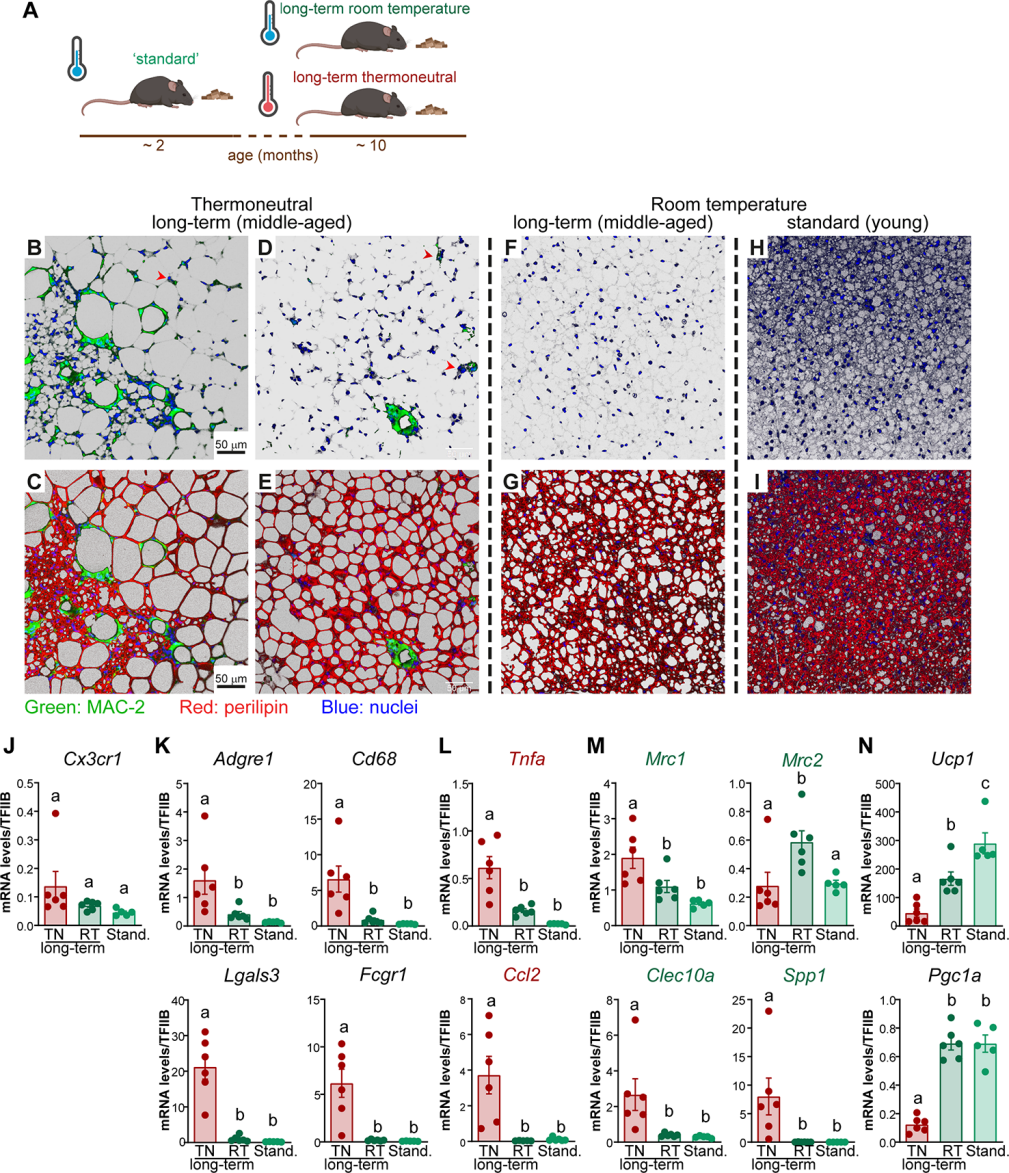
The effect of age on macrophage accumulation in brown fat. **(A)** Schematic representation of experimental groups. **(B**–**I)** Representative confocal images of IBAT from long-term thermoneutral mice **(B**–**E)**, ~ 11 month old mice housed at room temperature **(F, G)** and standard mice **(H**–**I)** stained for MAC-2 (green), perilipin (red) and nuclei (blue) using immunohistochemistry. Scale bar 50 μm (also applies to **D–I**). **(J**–**N)** Gene expression levels of tissue-resident macrophage marker gene **(J)**, general macrophage marker genes **(K)**, M1 macrophage marker genes **(L),** M2 macrophage marker genes **(M)** and thermogenesis-related genes **(N)** in IBAT of long-term thermoneutral mice (~ 11 month old) (n = 6), in IBAT of ~ 11-month-old mice housed at room temperature (n = 6) and in IBAT of ~ 2-month-old mice housed at room temperature (n = 5). The tissues were analyzed in parallel. Values are means ± S.E. Statistical significance by one-way ANOVA with Tukey post-test (different letters denote groups that are significantly different from each other, with p < 0.05).

Based on the gene expression analysis, which indicated pronounced macrophage accumulation in brown fat, we examined the morphology, frequency and distribution of macrophages in the tissue. Macrophages were visualized with an anti-MAC-2 antibody (green); MAC-2, also known as galectin-3 or LGALS3, is a lectin that mediates macrophage phagocytic and inflammatory responses (15, 16). The (brown) adipocytes were visualized with an anti-perilipin antibody (red) (**Figures 3B–I**). For clearer visualization of macrophages, in the upper panels only MAC-2 and nuclei are presented (here and throughout the paper).

In agreement with earlier reports, under thermoneutral conditions, the majority of brown adipocytes attained a unilocular appearance (**Figures 3C, E**) and e.g. (2, 6, 8, 17–19). Multilocular adipocytes were sporadically observed, usually organized in islands surrounded by unilocular adipocytes (**Figure 3C** and (2)).

In IBAT of long-term thermoneutral mice, macrophages were readily detected (**Figures 3B–E**), principally in accordance with earlier studies (20). In white adipose tissue, the macrophages have been reported to predominantly localize around dead adipocytes and fuse into multinucleate giant cells named crown-like structures (21, 22). Similarly, the vast majority of macrophages were aggregated into crown-like structures (**Figures 3B–E**), while a few macrophages were scattered within the tissue as solitary macrophages (some of these are depicted with red arrowheads). Macrophages within brown fat, similarly to those in WAT (7, 21), displayed nonrandom distribution. Some regions of IBAT were heavily infiltrated (**Figures 3B, C**); crown-like structures, as well as solitary macrophages, were readily detected in both unilocular (**Figures 3D, E**) and multilocular (**Figures 3B, C**) areas of the tissue. However, some regions of the tissue, mostly unilocular, contained very few or no macrophages (**Figures 3D, E**). Also, remarkable individual differences in the abundance of macrophages accumulating in brown fat were observed; some animals appeared to be resistant, while some animals demonstrated a high propensity for macrophage accumulation (as those presented in **Figures 3B, C**).

### The Effect of Age on Macrophage Accumulation in Brown Fat

Long-term thermoneutral mice were 9-11 months old when analyzed, as opposed to 2 months for standard mice. Thus, the transcriptome remodeling observed in mice exposed to thermoneutrality for a prolonged time could hypothetically be influenced by increased age. To discriminate the effects of age and temperature, mice that were of a similar age to the long-term thermoneutral mice (about 10 months old), but that were raised and maintained under standard conditions (21°C), were analyzed in parallel to the long-term thermoneutral and standard mice (schematically depicted in **Figure 3A**). As shown in **Figures 3F–I**, the brown fat from either middle-aged mice acclimated to room temperature (**Figures 3F, G**) or from standard mice (**Figures 3H, I**) was not visibly infiltrated with macrophages. This thus strongly indicates that age is not the determinative factor for macrophage accumulation in brown fat upon prolonged exposure to thermoneutrality. However, age had a profound effect on the morphology of brown adipocytes: although the cells were generally multilocular, the lipid droplets in the brown adipocytes of the middle-aged mice were notably larger than in the brown adipocytes found in the brown fat of standard (thus young) mice (**Figures 3G, I**).

Additionally, we determined the expression levels of macrophage marker genes (tissue resident (**Figure 3J**), general (**Figure 3K**), pro-inflammatory (M1 subtype) (**Figure 3L**), and anti-inflammatory (M2 subtype) (**Figure 3M**)) in the brown fat of the three groups of mice: middle-aged acclimated to thermoneutrality or to room temperature, and standard mice (young and room temperature). The expression levels of almost all macrophage marker genes were much lower in brown fat of mice acclimated to room temperature (middle-aged and standard) than in brown fat of thermoneutral mice (*Cx3cr1* and *Mrc2* were exceptions). Thus, the macrophage marker gene expression data were in agreement with the morphological observations.

To quantitatively determine the significance of each of the two factors – the age and the temperature – we performed a multiple linear regression analysis. In addition to macrophage marker genes, we included in the analysis *Ucp1* and *Pgc1a*, two genes related to thermogenesis; their expression levels were significantly higher in brown fat of mice acclimated to room temperature (**Figure 3N**). The multiple regression analysis (**Supplementary Table 2**) demonstrated that the expression levels of all examined genes were significantly influenced by the temperature (tissue-resident macrophages marker gene *Cx3cr1* was an exception). Age had an effect only on the expression of *Ucp1* and *Mrc2*. Thus, based on cellular and molecular analyses, temperature (thermoneutrality) was the factor most associated with macrophage accumulation in brown fat.

### Time Course of Thermoneutrality-Driven Macrophage Accumulation in Brown Fat

**Figure 4:**
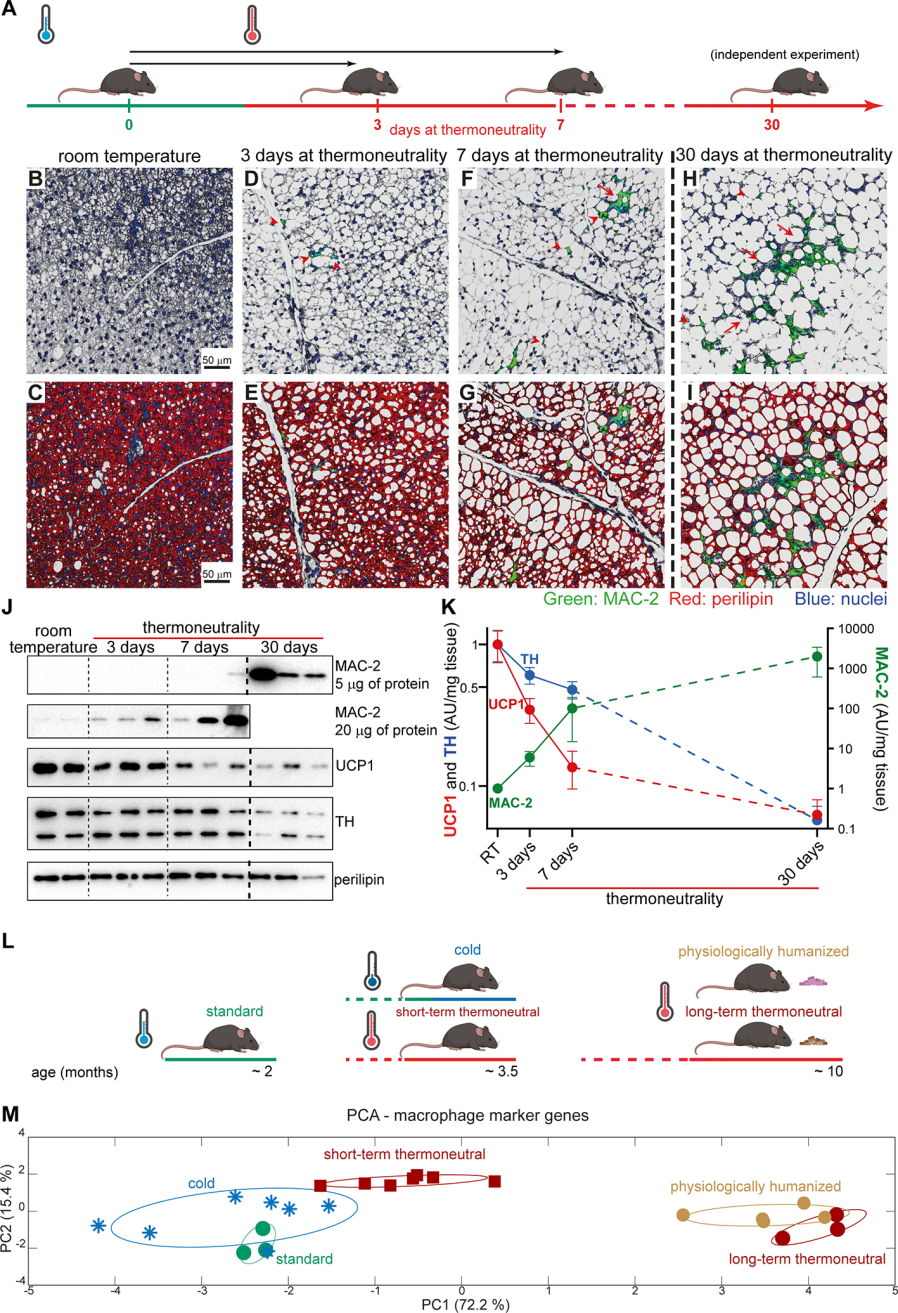
Time course of thermoneutrality-driven macrophage accumulation in brown fat. **(A)** Schematic representation of experimental groups analyzed in **(B–K)**. Mice acclimated to room temperature were exposed to thermoneutrality for 3 (n = 3), 7 (n = 3) or 30 (n = 3) days, or remained at room temperature (n = 2). Mice exposed to thermoneutrality for 30 days were from a different cohort (indicated by stippled line); the samples from the two cohorts were analyzed in parallel. **(B**–**I)** Representative confocal images of IBAT, stained for MAC-2 (green), perilipin (red) and nuclei (blue). Scale bar 50 μm (applies also to **D–I**). **(J)** Representative Western blots of MAC-2, UCP1, TH and perilipin in IBAT. Perilipin and UCP1 were detected using 5 μg of total protein. TH was detected using 20 μg of total protein. Stippled lines serve as visual separators between groups and do not indicate separate blots. **(K)** MAC-2, UCP1, and TH protein density in IBAT. The values in IBAT of mice acclimated to room temperature were set to 1.0, and the levels in IBAT of mice exposed to thermoneutrality for 3, 7, or 30 days were expressed relative to this value. Note logarithmic y-axes. Values are means ± S.E. **(L)** Schematic representation of experimental groups analyzed in **(M)**. Standard, long-term thermoneutral and physiologically humanized mice are those presented in **Figure 1A**. Thermoneutral mice were acclimated to thermoneutrality (30°C) for 6 weeks. “Cold” mice were acclimated to cold by being exposed first to 18°C for 1 week and then to 4°C for the following 4 to 5 weeks. **(M)** Principal component analysis of macrophage marker genes (*Cx3Cr1*, *Adgre1*, *Lgals3*, *Cd68*, *Fcgr1*, *Tnfa*, *Ccl2*, *Mrc1*, *Mrc2*, *Clec10a*, and *Spp1*) in the indicated samples (the samples were analyzed with RNA Sequencing). Each symbol represents one sample. Numbers in parentheses on the axes represent the proportion of data variance explained by each principal component. Note that to obtain uniform representation of variance over the graph surface, the axes were adjusted according to the percentage of variance explained by each of the components.

To reveal the dynamics of macrophage accumulation in brown fat upon exposure to thermoneutrality, mice acclimated to room temperature were exposed to thermoneutrality for 3, 7 or 30 days (schematically depicted in **Figure 4A**). Brown fat from mice acclimated to room temperature was not visibly infiltrated with macrophages (**Figures 4B, C**). However, already after 3 days exposure to thermoneutrality, solitary macrophages were detected (red arrowheads) (**Figures 4D, E**). After 7 days at thermoneutrality, both crown-like structures (red arrow) and solitary macrophages (red arrowheads) were observed in the tissue (**Figures 4F, G**); these data are principally in agreement with (20). In brown fat of mice that were exposed to thermoneutrality for 30 days, large areas of the tissue were infiltrated by macrophages predominantly organized into crown-like structures (red arrows) (**Figures 4H, I**).

The amount of macrophages in the tissue, quantitatively determined using immunoblot analysis for MAC-2 (**Figure 4J**, the top two panels and **Figure 4K**, green symbols/line), demonstrated a steady increase with time at thermoneutrality and thus fully reflected the results obtained by immunohistochemistry.

Exposure to thermoneutrality also had a profound and rapid effect on the morphology of the tissue itself. Lipid content in the tissue and the number of unilocular brown adipocytes increased notably even after only 3 days at thermoneutrality (compare **Figure 4C** and **Figure 4E**) and gradually increased with time at thermoneutrality (**Figures 4C, E, G, I**). In mice that were exposed to thermoneutrality for 30 days, nearly all adipocytes in brown fat had attained a unilocular appearance (**Figure 4I**).

**Figure 5:**
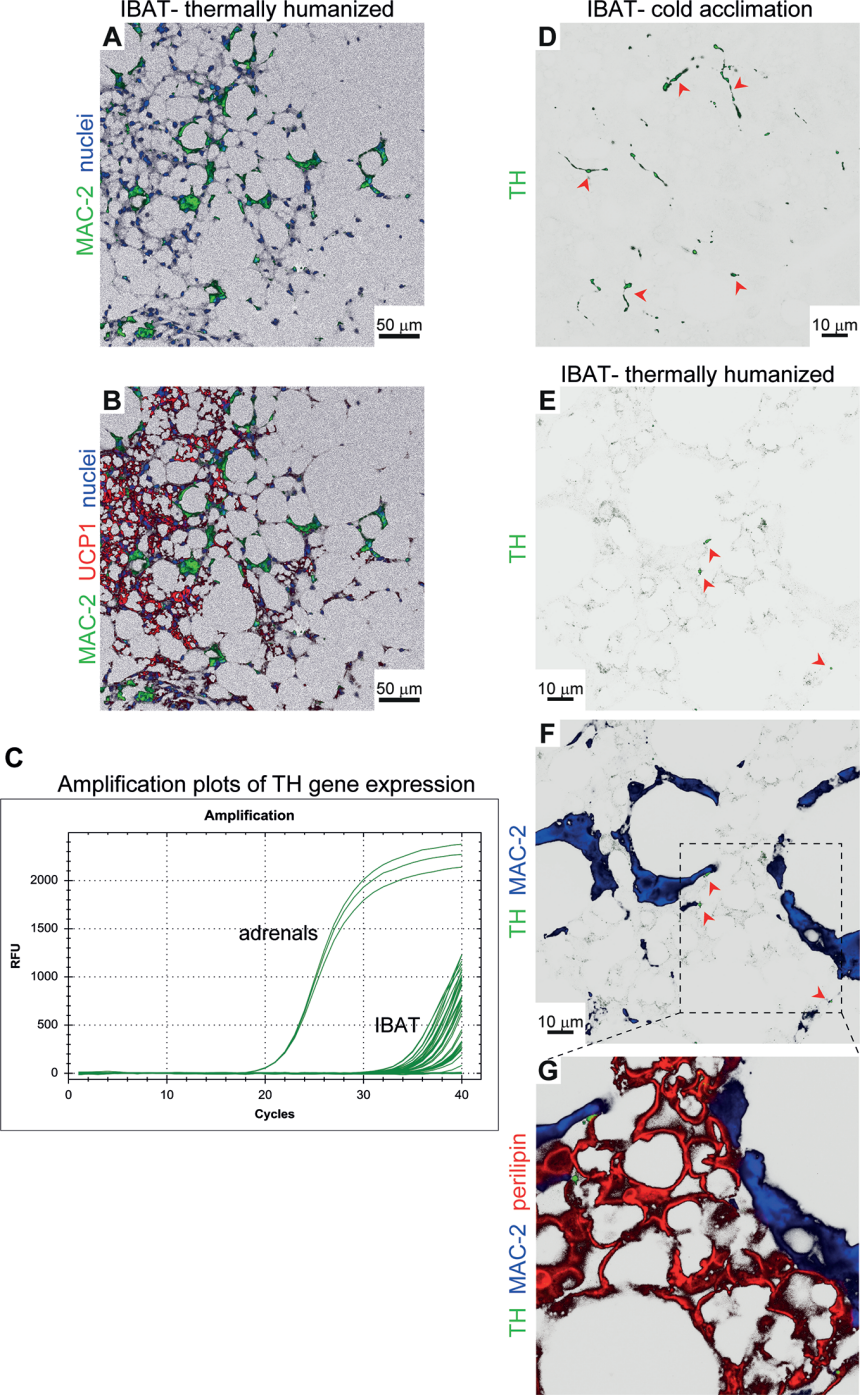
Brown-fat macrophages do not synthesize catecholamines. **(A**, **B)** Representative confocal images of IBAT from long-term thermoneutral mice stained for MAC-2 (green), UCP1 (red) and nuclei (blue) using immunohistochemistry. Scale bar 50 μm. **(C)** Amplification plots of tyrosine hydroxylase (TH) gene expression in IBAT of long-term thermoneutral and physiologically humanized mice. An equal amount of adrenal cDNA was amplified in parallel. **(D–G)** Representative confocal images of IBAT from cold-acclimated **(D)** and long-term thermoneutral **(E–G)** mice stained for tyrosine hydroxylase (TH) (green), MAC-2 (blue) and perilipin (red) using immunohistochemistry. Scale bar 10 μm (does not apply to **G**).

The appearance of brown fat and the expression of thermogenesis-related genes in the tissue are mainly determined by the degree of sympathetic stimulation of the tissue (1). The rate-limiting enzyme in catecholamine biosynthesis is tyrosine hydroxylase (23). Tyrosine hydroxylase is transcribed and translated in the neuronal bodies and the protein is then transported to the nerve endings in the tissue (as visualized in **Figure 5D** and e.g. (24)). Tyrosine hydroxylase protein levels in the tissue reflect the ability of the tissue to synthesize catecholamines. As shown in **Figures 4J, K** (blue symbols/line), during the exposure to thermoneutrality, tyrosine hydroxylase protein levels in the tissue diminished, indicating a decrease of sympathetic nervous activity. The levels of UCP1 protein also decreased rapidly with time at thermoneutrality (**Figures 4J, K**, red symbols/line). Thus, the appearance of macrophages in brown fat coincides with the cessation of its thermogenic activity.

Brown fat in response to altered thermogenic demands attains a new thermogenic recruitment state within about 3 to 4 weeks. To examine whether the macrophages that accumulated in brown fat upon 30-day acclimation to thermoneutrality also reached a new steady state, we performed PCA of macrophage marker genes in brown fat of mice acclimated to thermoneutrality for about 6 weeks, compared with mice acclimated to thermoneutrality for a very long time or mice acclimated to subthermoneutral temperatures (schematically depicted in **Figure 4L**). Brown fat from standard mice (green circles) and from mice acclimated to 4°C (blue snowflakes) displayed remarkable similarity (**Figure 4M**). Brown fat samples from mice that were acclimated to thermoneutrality for six weeks (brown squares) formed a distinct group. However, the effect of short-term thermoneutrality on macrophage molecular signature was rather small. In contrast, brown fat samples from the two groups of mice that were acclimated to thermoneutrality for a very long time (long-term thermoneutral (brown circles) and physiologically humanized (ochre circles)) formed a distinct cluster positioned far from the other samples; again no effect of an energy-rich diet was observed. Thus, the thermogenic steady state of the tissue was not paralleled by the macrophage steady state, i.e. macrophage content/activity in the tissue continued to change with time at thermoneutrality.

### No Norepinephrine-Producing Capacity of Brown Adipose Tissue Macrophages

In brown fat of physiologically humanized mice, only multilocular adipocytes, usually found in islands surrounded by unilocular adipocytes, are clearly UCP1-positive (2). This indicates that these regions of the tissue could have been exposed to higher adrenergic stimulation. Simultaneous analysis of UCP1 and MAC-2 in brown fat of long-term thermoneutral mice revealed that areas composed of multilocular UCP1-positive brown adipocytes were heavily infiltrated by macrophages (**Figures 5A, B**). It may therefore be suggested that these macrophages could have influenced the thermogenic gene expression in the tissue through release of norepinephrine or other catecholamines (25). The tyrosine hydroxylase found in the sympathetic nerves in the tissue is nearly entirely synthesized in the nerve cell bodies found in the sympathetic chain (or corresponding ganglion), and tyrosine hydroxylase mRNA is therefore practically absent in the nerve fibers within the brown adipose tissue itself (26). Accordingly, if macrophages in the tissue should be able to synthesize norepinephrine, this would require that the tyrosine hydroxylase gene was readily expressed in these macrophages so that tyrosine hydroxylase protein could be synthesized within the macrophages. We therefore examined whether tyrosine hydroxylase mRNA was present in the tissue. We verified that we could identify tyrosine hydroxylase mRNA in the adrenal gland. However, we were unable to obtain a satisfactory signal for tyrosine hydroxylase mRNA in IBAT from thermoneutral mice (Ct values above 35) (**Figure 5C**). The essential absence of tyrosine hydroxylase mRNA demonstrated that there were no cells capable of synthesizing tyrosine hydroxylase protein in the tissue. This means that the macrophages do not have the ability to secrete catecholamines. Thus, the apparent close spatial association between the assembly of macrophages and the presence of UCP1 was not due to macrophage-associated norepinephrine synthesis.

In tissue sections of IBAT from mice acclimated to cold, a situation characterized by dense sympathetic innervation (27), we identified tyrosine hydroxylase protein in dotted foci within fiber-like structures (**Figure 5D**). However, such structures were not detected in IBAT of long-term thermoneutral mice; only few dotted foci were observed (red arrowheads (**Figures 5E, F**)) that were closely associated with brown adipocytes (**Figure 5G**). Importantly, no tyrosine hydroxylase immunoreactivity was detected within macrophages (blue). Thus, the macrophages infiltrating brown fat of thermoneutral mice were unable, by paracrine secretion of catecholamines, to influence the appearance of multilocular brown adipocytes and the expression of thermogenesis-related genes and thermogenesis in the tissue (as is also the case under subthermoneutral conditions (28).

### Thermoneutrality-Driven Macrophage Accumulation in Brown Fat Is Reversed by Exposure to Cold

**Figure 6:**
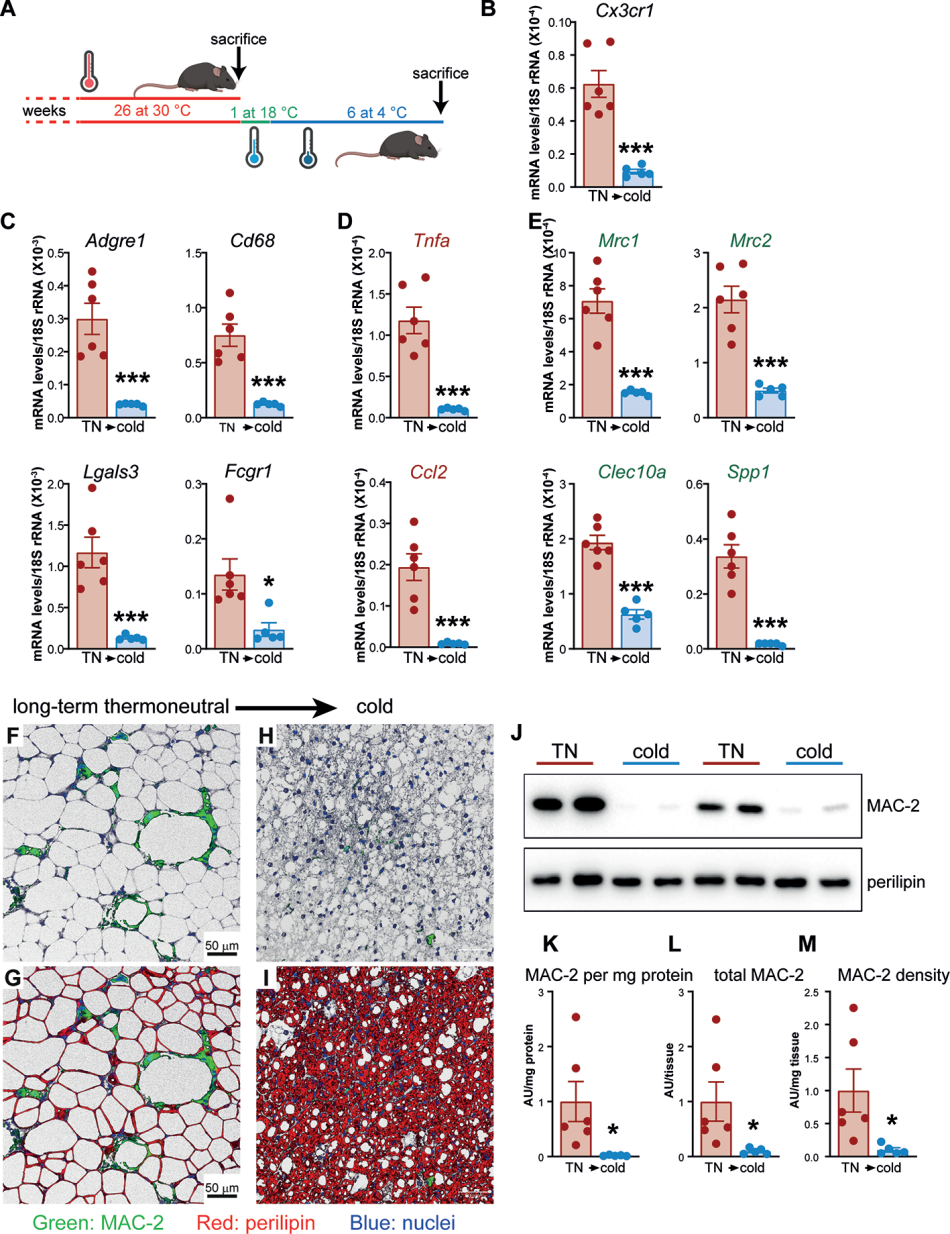
Thermoneutrality-driven macrophage accumulation in brown fat is reversed upon acclimation to cold. **(A)** Schematic representation of experimental groups. **(B–E)** Gene expression levels of tissue-resident macrophage marker gene **(B)**, general macrophage marker genes **(C)**, M1 macrophage marker genes **(D)** and M2 macrophage marker genes **(E)** in IBAT of long-term thermoneutral mice (n = 6) and in IBAT of cold-acclimated, previously long-term thermoneutral mice (n = 5). Values are means ± S.E. ***P < 0.001, significant difference between temperatures by Student’s unpaired t-test. **(F–I)** Representative confocal images of IBAT from long-term thermoneutral mice **(F, G)** and cold-acclimated, previously long-term thermoneutral mice **(H, I)**, stained for MAC-2 (green), perilipin (red) and nuclei (blue) using immunohistochemistry. Scale bar 50 μm (applies also to H and I). **(J)** Representative Western blots of MAC-2 and perilipin in IBAT of the mice as in **(B–I)**. **(K–M)** MAC-2 protein levels **(K)**, MAC-2 protein content **(L)** and MAC-2 protein density **(M)** in IBAT of the mice as in **(B–I)**. The values in IBAT of long-term thermoneutral mice were set to 1.0, and the levels in IBAT of cold-acclimated, previously long-term thermoneutral mice were expressed relative to this value. Values are means ± S.E. *P < 0.05, significant difference between temperatures by Student’s unpaired t-test.

To elucidate whether the profound accumulation of macrophages occurring in brown fat of long-term thermoneutral mice could be reversed, we examined macrophage content in brown fat of long-term thermoneutral mice subsequently acclimated to cold (schematically depicted in **Figure 6A**). The expression levels of macrophage marker genes in brown fat of long-term thermoneutral mice subsequently acclimated to cold were lower than in brown fat of long-term thermoneutral mice (**Figures 6B–E**), indicating a decrease in the number of macrophages in the tissue. The crown-like structures, regularly observed in brown fat of long-term thermoneutral mice (**Figures 6F, G**) were not present in the brown fat of the mice subsequently acclimated to cold (**Figures 6H, I**); only solitary macrophages were occasionally observed.

**Figure 7:**
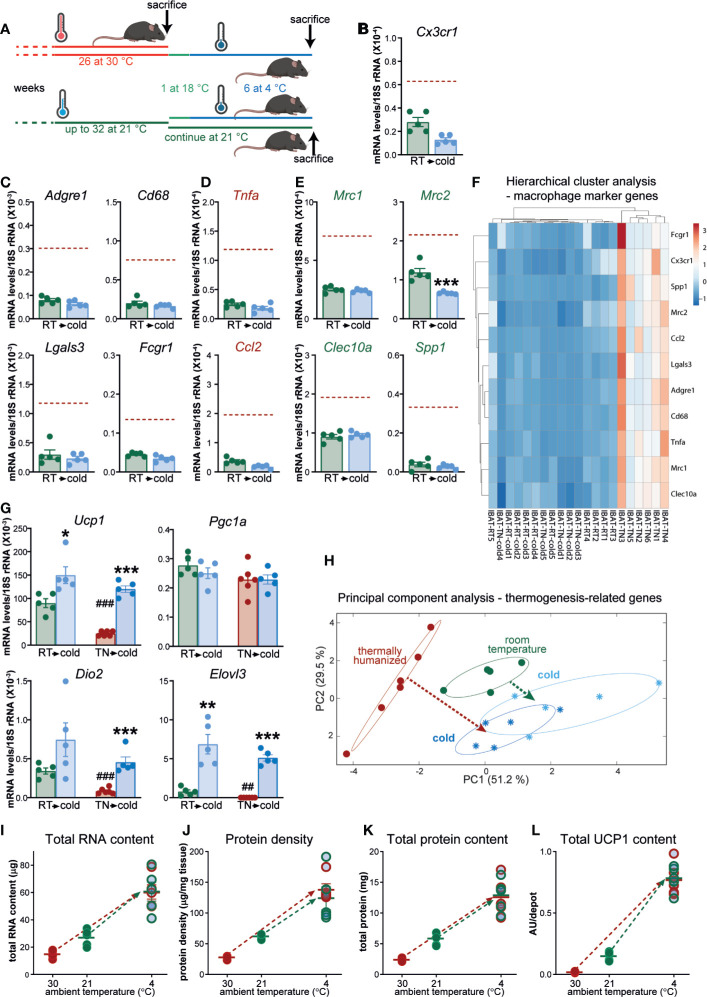
Macrophage accumulation in brown fat upon prolonged exposure to thermoneutrality does not affect the tissue’s thermogenic recruitment competence. **(A)** Schematic representation of experimental groups. **(B–E)** Gene expression levels of tissue-resident macrophage marker gene **(B)**, general macrophage marker genes **(C)**, M1 macrophage marker genes **(D)** and M2 macrophage marker genes **(E)** in IBAT of mice housed at room temperature (n = 5) and in IBAT of mice acclimated to cold, previously housed at room temperature (n = 5). Brown stippled line denotes mean values in long-term thermoneutral mice (presented in **Figures 6B–E**). For clarity, scales in **Figures 6B–E** and **(B–E)** are equal. Values are means ± S.E. ***P < 0.001, significant difference between temperatures by Student’s unpaired t-test. **(F)** Hierarchical cluster analysis of the expression levels of the genes presented in **Figures 6B–E** and **(B–E)**. The heatmap illustrates relative gene expression. Rows are centered; unit variance scaling is applied to rows. Both rows and columns are clustered using Euclidean distance and average linkage. **(G)** Expression levels of thermogenesis-related genes in IBAT of long-term thermoneutral mice (presented in **Figure 6**) and mice of similar age housed at room temperature [presented in **(B–E)**], both acclimated (or not) to cold. Values are means ± S.E. *P < 0.05; **P < 0.01; ***P < 0.001, significant difference between cold and antecedent acclimation temperature (room temperature or thermoneutral temperature) by Student’s unpaired t-test. ^##^P < 0.01; ^###^P < 0.001, significant difference between thermoneutral temperature and room temperature by Student’s unpaired t-test. **(H)** Principal component analysis of thermogenesis-related genes (*Ucp1*, *Pgc1a*, *Dio2*, *Elovl3*, *Gpd2*, Gyk, *Fgf21*, *Cox7a1*, *Prdm16*, and *Cidea*) in the indicated samples (the samples were analyzed with quantitative real-time PCR). Each symbol represents one sample. Numbers in parentheses on the axes represent the proportion of data variance explained by each principal component. Note that to obtain uniform representation of variance over the graph surface, the axes were adjusted according to the percentage of variance explained by each of the components. **(I–L)** Total RNA content **(I)**, protein density **(J)**, total protein content **(K)** and total UCP1 content **(L)** in one lobe of IBAT of the mice presented in **Figure 6** and **(B–G)**. Values are means ± S.E. Where not visible, the error bars are smaller than the symbols.

To quantitatively determine macrophage content in IBAT of the two groups of mice, we quantified MAC-2 protein amounts (**Figure 6J**, upper panel). MAC-2 protein levels varied markedly in brown fat of long-term thermoneutral mice (**Figure 6K**). Importantly, however, cold acclimation led to a marked reduction of MAC-2 protein levels in brown fat (**Figures 6J, K**). The total MAC-2 protein amount in the IBAT (**Figure 6L**) was calculated by multiplying the MAC-2 protein levels expressed per mg tissue protein (**Figure 6K**) with the total protein content of the tissue (**Figure 7K**). Even though the total IBAT protein content was increased more than 5 times upon cold acclimation, the total MAC-2 content was still strikingly lower in brown fat of cold-acclimated than in brown fat of long-term thermoneutral mice (**Figure 6L**). The tissue density of MAC-2, approximating the number of macrophages per mg tissue, was also much lower in IBAT of cold-acclimated mice (**Figure 6M**). Thus, brown fat, initially heavily infiltrated with macrophages due to prolonged exposure to thermoneutrality, was principally depleted of macrophages when thermogenically (re)activated.

### Macrophages That Accumulate in Brown Fat Upon Prolonged Exposure to Thermoneutrality Do Not Affect Its Competence for Thermogenic Recruitment

In long-term thermoneutral mice, brown fat remains competent to be recruited upon chronic exposure to cold (2). However, in view of the highly abundant macrophages in brown fat of long-term thermoneutral mice, the question may be asked whether the macrophages could negatively (or positively) influence the level of thermogenic recruitment.

The above question can be resolved by comparing the degree of brown-fat thermogenic recruitment in long-term thermoneutral mice with that observed in tissue that has never contained a large number of macrophages. Brown fat of mice housed at room temperature is principally devoid of macrophages (see above). Therefore, by directly comparing the cold-induced brown-fat recruitment levels in the two groups of mice of similar age but with initially different levels of macrophages in brown fat — long-term thermoneutral and lifelong housed at room temperature (schematically depicted in **Figure 7A**) — we aimed to reveal the potential effect of macrophages on this adaptive process.

We first verified the disparate abundance of brown fat macrophages in these experimental sets of long-term thermoneutral mice and middle-aged mice housed at room temperature. The expression levels of all examined macrophage marker genes were significantly lower in the brown fat of the mice acclimated to room temperature than in that of long-term thermoneutral mice (**Figures 7B–E**, green bars vs. brown stippled line). Thus, the results obtained in the original experimental sets of long-term thermoneutral mice and middle-aged mice housed at room temperature (see above, **Figure 3**) were fully reproduced in these new sets of mice. Particularly large differences were observed in the expression levels of general (**Figure 7C**) and M1-selective (**Figure 7D**) macrophage marker genes. Notably, in the brown fat of the mice acclimated to room temperature, the expression levels of macrophage marker genes were not further diminished upon acclimation to cold (**Figures 7B–E**).

To obtain an overview of the expression levels of the selected macrophage marker genes in the brown fat of the four groups of mice, the results presented in **Figures 6B–E** and in **Figures 7B–E** were visualized as a heatmap on the basis of hierarchical clustering (**Figure 7F**). Brown fat samples from long-term thermoneutral mice comprised one cluster. Brown fat samples from long-term thermoneutral mice subsequently acclimated to cold were indistinguishable from the samples of the other two groups of mice that were only exposed to subthermoneutral temperatures, which all together comprised the second cluster, further emphasizing that the brown fat from long-term thermoneutral mice was the only tissue examined that was heavily infiltrated by macrophages.

We then examined the magnitude of the induction of genes related to thermogenesis in response to cold stimulus in brown fat devoid of macrophages (from mice housed at room temperature) or rich in macrophages (from long-term thermoneutral mice). The initial mRNA levels of thermogenesis-related genes were higher in brown fat of mice housed at room temperature (**Figure 7G**, green bars/symbols) than in brown fat of long-term thermoneutral mice (**Figure 7G**, brown bars/symbols). Upon acclimation to cold, the induction of thermogenesis-related genes was observed in both groups (*Pgc1a* was an exception). Importantly, cold-induced mRNA levels of thermogenesis-related genes were very similar in the two groups of mice (**Figure 7G**, dark blue vs. light blue bars/symbols).

To obtain an overview of the dynamic changes of the brown fat thermogenic program in the two groups of mice upon acclimation to cold, we performed PCA using a set of genes related to thermogenesis (in addition to *Ucp1*, *Pgc1a*, *Dio2*, and *Elovl3*, also *Gpd2*, *Gyk*, *Fgf21*, *Cox7a1*, *Prdm16*, and *Cidea* were included (**Figure 7H**). IBAT samples from long-term thermoneutral mice (brown circles) formed a distinct cluster positioned far from the other samples. Cold acclimation of long-term thermoneutral mice led to marked alterations in the brown fat thermogenic program (**Figure 7H**, dark blue snowflakes), indicated by a brown arrow. Importantly, brown fat thermogenic programs in the two groups of cold-acclimated mice displayed remarkable similarity (**Figure 7H**, dark and light blue snowflakes) despite their different thermal history. Notably, the brown fat samples from animals housed at room temperature (green circles) were positioned much closer to the samples from the cold-acclimated than to the samples from the long-term thermoneutral mice. As brown fat at 21°C is already essentially fully differentiated (but not fully recruited), it is not surprising that no large alterations in the brown fat thermogenic gene expression program were observed when these mice were acclimated to cold (indicated by the green arrow) [see also (29)].

The cold-induced recruitment parameters of the brown fat in the long-term thermoneutral and in the mice that were lifelong housed at room temperature (partly reported in the Supplemental data of our recent publication (2)), are presented here (**Figures 7I–L**) in an edited comparative form, in order to highlight both the differences and similarities between these two groups of mice before and after acclimation to cold. The initial values of all examined brown-fat parameters (**Figures 7I–L**) were higher in mice housed at room temperature than in long-term thermoneutral mice (green and brown circles, respectively). However, following cold acclimation, all examined parameters were significantly increased in both groups of mice (indicated by the green and brown arrows) (**Figures 7I–L**). Cold-induced levels of all four examined parameters were nearly identical in the two groups of mice.

Thus, the brown fat thermogenic profile attained upon acclimation to cold was determined by the actual rather than by the antecedent acclimation temperature and also did not correlate to the initial number of macrophages in the tissue. Importantly, brown fat of long-term thermoneutral mice, although heavily infiltrated by macrophages, was fully competent to attain the greatest possible recruitment state.

## Discussion

In the present investigation, we demonstrate that prolonged exposure to thermoneutrality invokes abundant accumulation of macrophages in brown fat; neither an energy-rich diet nor increased age led to any significant effect on this accumulation. These numerous macrophages are primarily organized into crown-like structures and are in no way detrimental for subsequent cold-induced brown adipose tissue recruitment.

### Brown Fat Is Not Resistant to Macrophage Accumulation

In adipose tissues, macrophages have been mostly studied in the context of obesity. Massive infiltration of macrophages in white adipose tissue (WAT) under obesogenic conditions is an established phenomenon (13, 14). Under such conditions, macrophages accumulate in visceral adipose depots, aggregate around dead or dying adipocytes and form multinucleate crown-like structures (21, 22). Accumulation of macrophages in BAT and their impact on BAT function have been studied much less extensively, and mainly in animals fed an energy-rich diet and maintained at standard housing (and thus physiologically semi-cold) temperatures (30–36). Low (30) or no (32) inflammation and macrophage infiltration in BAT compared with WAT have implicated BAT resistance towards obesity-induced inflammation. However, in comparison with BAT from mice fed a chow diet, BAT from mice fed a sufficiently sustained obesogenic diet did display significantly higher expression levels of inflammatory marker genes (30, 33). The only earlier study that examined the effects of diet and housing temperature on the development of metabolic inflammation in mice, demonstrated a significant macrophage accumulation in BAT at both standard and thermoneutral temperatures dependent on an energy-rich diet (31). Here, on the contrary, we demonstrate that macrophage accumulation in the BAT of thermoneutral mice is not dependent on an energy-rich diet. However, our study was performed on mice that were fed an energy-rich diet containing 45% energy from fat for at least six months, as opposed to (31) in which mice were fed an energy-rich diet containing 60% energy from fat for maximum three months. Importantly, despite the differences observed, these earlier studies and our present findings, together, clearly demonstrate that BAT can accumulate a remarkably high amount of macrophages.

### The “Identity” of Macrophages in Thermoneutral Brown Fat

In tissues, cells of the monocyte-macrophage lineage mature and are activated in a dynamic response to distinct environmental cues to acquire specialized functional phenotypes: classical (proinflammatory) M1 (in response to TLR ligands and IFN-γ) or alternative (anti-inflammatory) M2 (in response to IL-4/IL-13) (37–39). The paradigm of the monocyte-macrophage lineage polarization into M1 or M2 subtypes of macrophages, essentially based on *in vitro* results, is an operationally useful but very simplified descriptor of the functional plasticity of these cells (40). *In vivo*, coexistence of cells in different activation states, as well as unique or mixed phenotypes, have been observed, reflecting dynamic changes and complex tissue-derived signals (39, 41).

In the present study, we demonstrate that the general, as well as M1-selective and M2-selective, macrophage marker genes are readily detected in BAT of thermoneutral mice. Since the analysis was performed at the whole tissue level and not on the fraction of freshly isolated and purified brown-fat macrophages, any molecular/functional classification of these cells is currently not possible. Given that the majority of macrophages found in brown fat of thermoneutral mice are organized into multinucleate giant crown-like structures, the feasibility of developing procedures for their isolation and the reliability of downstream analyses, particularly those performed at a single-cell level (such as FACS analysis), seem insurmountable. A possibility to circumvent this challenge could be to perform the analysis *in situ*. The recent development of sensitive and versatile *in situ* hybridization methods opens a possibility for accurate molecular characterization of brown fat macrophages *in situ*, regardless of whether they are organized into crown-like structures or as solitary macrophages. We expect that these future studies will reveal and clarify the true identity of macrophages in brown fat of thermoneutral mice (e.g. whether they are polarized into M1 and M2 subtypes or rather display multiple complex phenotypes (such as those mentioned below)).

### No Effect of Macrophages on Brown Fat Thermogenic Function

The major regulator of both brown fat thermogenic activity and brown fat recruitment is norepinephrine (NE) released from sympathetic nerves (1). However, in recent years, it has been suggested that the availability of NE and thus BAT thermogenic activity can be modulated by macrophages occurring in the tissue (25, 42, 43).

The classical dogma that NE in brown fat is of only sympathetic origin was challenged by Nguyen et al. (25) who suggested that alternatively activated macrophages are another important source of catecholamines and are required for adaptive thermogenesis. In a later study, in which we participated, it was demonstrated that the macrophages occurring in brown fat of mice acclimated to subthermoneutral temperatures (21°C and 4°C) do not synthesize catecholamines or contribute to adipose tissue adaptive thermogenesis (28). However, in those experiments, the macrophage density was low. Here we demonstrate that also under thermoneutral conditions, the vast amount of macrophages infiltrating brown fat do not affect the tissue through a paracrine secretion of catecholamines. However, macrophages may play a homeostatic role in the control of tissue innervation (44).

A NE-degrading and thus thermogenesis-regulatory function has been ascribed to specialized macrophages within BAT and WAT (42, 43). These adipose-specific NE-degrading macrophages are increased in number in obese states (42) or are activated in aged animals (43). However, the brown fat from long-term thermoneutral mice (as well as from physiologically humanized mice (2)) is fully competent to achieve the greatest possible recruitment state upon exposure to chronic cold (4°C) (see **Figure 7**). Thus, macrophages of any type, possibly including those specialized in uptake and degradation of catecholamines, have no persistent negative influence on thermogenic recruitment of brown fat, even in mice housed for a very long time under thermoneutral conditions.

### What Is the Function of Brown Fat Macrophages?

The conclusion that the macrophages abundantly present in brown fat of thermoneutral mice do not influence the competence for thermogenic recruitment of the tissue evidently raises the question of their physiological function. Brown adipose tissue is characterized by extraordinary plasticity and capacity to adapt its size, morphology and function to changing thermogenic demands (1). Thus, in response to prolonged exposure to subthermoneutral ambient temperatures, both brown fat hyperplasia and brown fat hypertrophy take place. On the contrary, when a cold-acclimated animal is transferred to thermoneutrality, there is an abrupt cessation of the thermogenic activity in the tissue and a new adaptation process starts. This adaptation involves the rapid degradation of proteins, including UCP1 (45, 46), a decrease in tissue cell number (45), a marked increase in the rate of apoptosis (47) and, as we demonstrate in the present study, the accumulation of macrophages in the tissue. Importantly, the appearance of macrophages in brown fat occurs rapidly after the animal has been transferred to thermoneutrality (**Figure 4**) and thus coincides with initiation of catabolic processes in the tissue and with accelerated death (apoptosis) of brown adipocytes (and other cell types). We therefore suggest that the macrophages found in thermoneutral brown fat execute their conventional (but probably not their only) function: phagocytosis and degradation of dead cells, dying cells and cellular debris. Brown fat macrophages thus orchestrate tissue remodeling and enable maintenance of metabolic homeostasis in the tissue. This is analogous to the situation in WAT, where adipose tissue inflammation is essential for healthy adipose tissue expansion (48) and where macrophages also mediate adipose tissue remodeling (12). However, the number of macrophages in brown fat remains markedly elevated (at variable levels) even after the tissue has reached a new thermogenic steady-state (after some months at thermoneutrality). Further exploration of this phenomenon will be needed to reveal its physiological cause.

## Data Availability Statement

The datasets presented in this study can be found in online repositories. The names of the repository/repositories and accession number(s) can be found below: https://www.ebi.ac.uk/arrayexpress/experiments/E-MTAB-7561/, E-MTAB-7561 https://www.ebi.ac.uk/arrayexpress/experiments/E-MTAB-9062/, E-MTAB-9062, https://www.ebi.ac.uk/arrayexpress/experiments/E-MTAB-7565/.

## Ethics Statement

The animal study was reviewed and approved by the Animal Ethics Committee of the North Stockholm region.

## Author Contributions

AF and NP designed the research. AF, JJ, FS, CS, and NP performed the experiments. JH provided essential materials. AF, JJ, FS, CS, and JH edited and revised the manuscript. NP wrote the manuscript and supervised the research. All authors contributed to the article and approved the submitted version.

## Funding

The authors acknowledge support from the Swedish Research Council (VR-2017-01379 and VR-2017-04715), the DFG grant HE3645/10-1, the DFG grant FI2476/1-1, the Novo Nordisk Foundation (NNF17C0027058), Magnus Bergvalls Stiftelse (2017-02199, 2018-02969 and 2019-03487), Carl Tryggers Stiftelse (CST 19: 282), Diabetesfonden (DIA 2018-381), European Union Collaborative projects ADAPT (EU201100) and DIABAT (EU278373) and Knut and Alice Wallenberg Foundation (WA2015-0009). The authors thank the Experimental Core Facility staff for breeding the mice and the Imaging Facility at Stockholm University for help with confocal microscopy. Schematic depictions were generated with biorender.com.

## Conflict of Interest

The authors declare that the research was conducted in the absence of any commercial or financial relationships that could be construed as a potential conflict of interest.
