# Supplementary material for: Thermoneutrality-Induced Macrophage Accumulation in Brown Adipose Tissue Does Not Impair the Tissue’s Competence for Cold-Induced Thermogenic Recruitment
Source: Front Endocrinol (Lausanne). 2020 Oct 30;11:568682. doi: 10.3389/fendo.2020.568682 (PMC7662517; doi:10.3389/fendo.2020.568682)
Supplement: Supplementary file 1 [file DataSheet_1.pdf]

Supplementary Figure 1

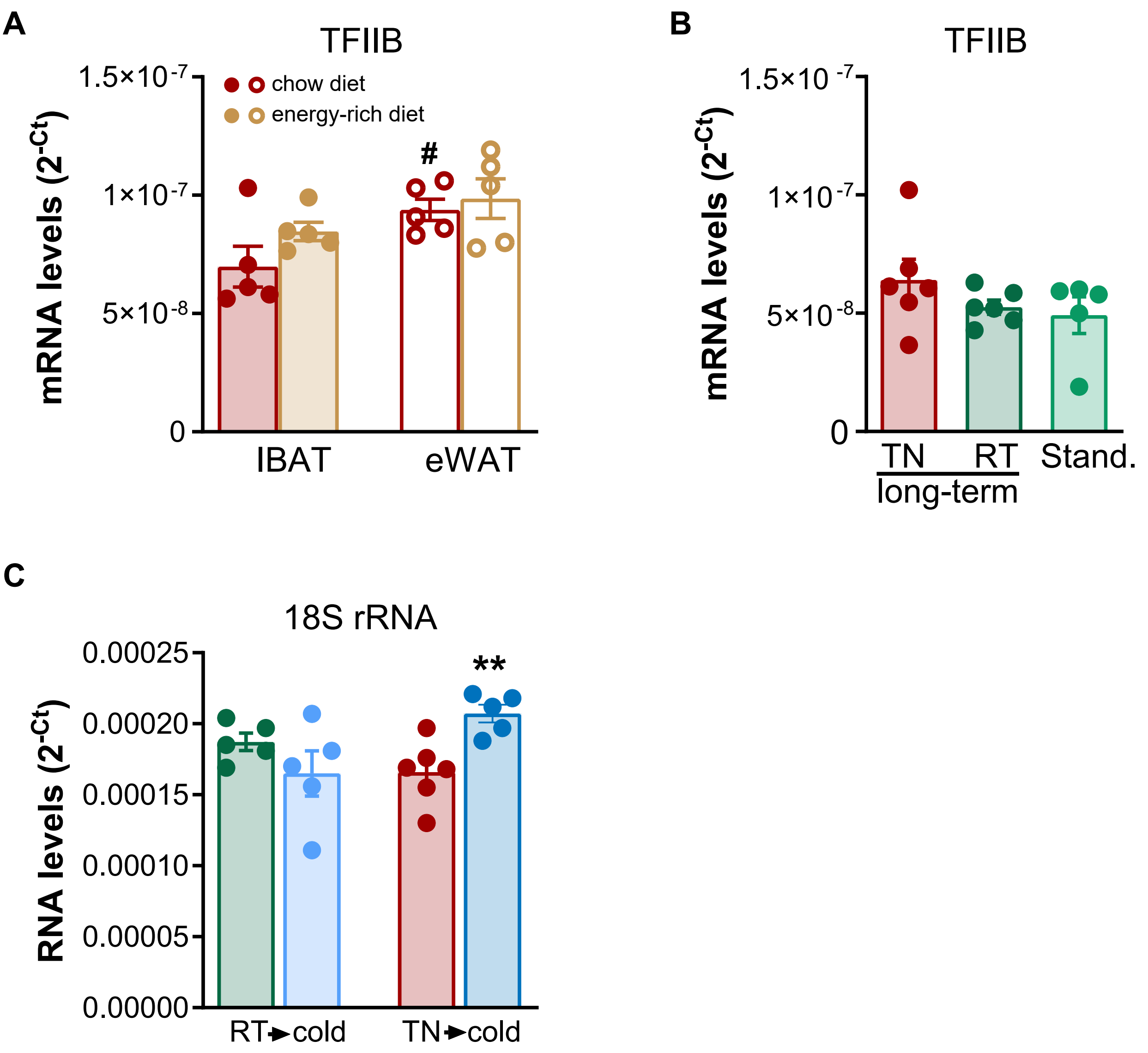

Supplementary Figure 1. Reference gene expression in adipose tissues. Values are antilog-transformed Ct values (2^{-Ct}) presented as means ± S.E.

(A) TFIIB gene expression levels in adipose depots presented in Figure 2. # $P < 0.05$; sigficant difference between IBAT and eWAT by Student's unpaired t-test.

(B) TFIIB gene expression levels in adipose depots presented in Figures 3B-F. No significant difference between groups by one-way ANOVA.

(C) 18S rRNA expression levels in adipose depots presented in Figures 6B-E, 7B-E and 7G.

** $P < 0.01$; significant difference between cold and thermoneutral temperature.

Supplementary Figure 2

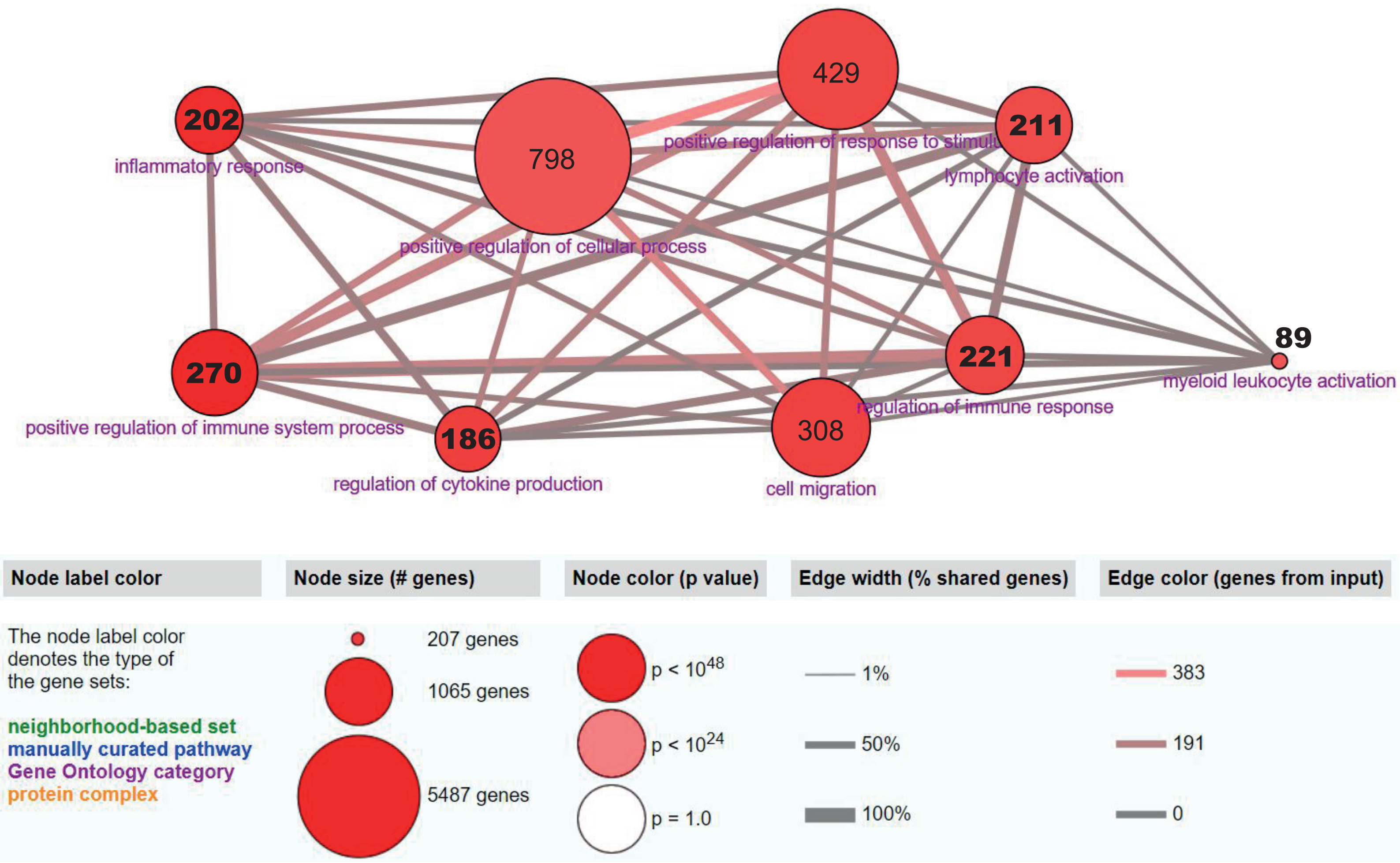

Supplementary Figure 2. Visualization of the top 9 Gene Ontology pathways upregulated in IBAT of both physiologically humanized and long-term thermoneutral mice versus mice exposed to standard conditions. The numbers within the nodes denote how many of the 2401 commonly upregulated genes are found in each node. Pathways related to inflammation are highlighted in bold.

Supplementary Table 1. Primer sequences

**Gene**	**Forward (5' - 3')**	**Reverse (5' - 3')**
Cx3cr1	AAGTTCCCTTCCCATCTGCT	CAAAATTCTCTAGATCCAGTTCAGG
Adgre1	GGAGGACTTCTCCAAGCCTATT	AGGCCTCTCAGACTTCTGCTT
Lgals3	GCCTACCCCAGTGCTCCT	GGTCATAGGGCACCGTCA
Cd68	CGGTGGAATACAATGTGTCCT	GATGAATTCTGCGCCATGA
Fcgr1	TGTGGCTTCTAACAACTCTGCT	ACAGCCTTGGTGGCATTAAC
Tnfa	TCTTCTCATTCCTGCTTGTGG	GGTCTGGGCCATAGAACTGA
Ccl2	CATCCACGTGTTGGCTCA	GATCATCTTGCTGGTGAATGAGT
Mrc1	CAAACTGGGGGAAAGGCTAT	TTGCCACCAATCACAACTACA
Mrc2	TACAGCTCCACGCTATGGATT	CACTCTCCCAGTTGAGGTACT
Clec10a	TGAGAAAGGCTTTAAGAACTGGG	GACCACCTGTAGTGATGTGGG
Spp1	CCCGGTGAAAGTGACTGATT	ATCTGGGTGGAGGCTGTAA
Pgc1a	GAAAGGGCCAAACAGAGAGA	GTAAATCACACGGCGCTCTT
Ucp1	GGCCTCTACGACTCAGTCCA	TAAGCCGGCTGAGATCTTGT
Dio2	CTGCGCTGTGTCTGGAAC	GGAATTGGGAGCATCTTCAC
Elovl3	GCCTCTCATCCTCTGGTCCT	TGCCATAAACTTCCACATCCT
TH	GCTACCGAGAGGACAGCATT	CACGGGCAGACAGTAGACC
18S	AGTCCCTGCCCTTTGTACACA	CGATCCGAGGGCCTCACTA
TFIIB	TGGAGATTTGTCCACCATGA	GAATTGCCAAACTCATCAAAACT

Supplementary Table 2. A multiple linear regression analysis P values and β values for influence of age and temperature on the expression of thermogenesis-related and macrophage marker genes

**Gene**	**P values**		**β values**	
	Age	temperature	age	temperature
Ucp1	< 0.01	< 0.01	-123.8	-120.7
Pgc1a	0.9918	< 0.001	0.00	-0.57
Cx3cr1	0.6107	0.1433	0.02	0.07
Adgre1	0.5440	< 0.05	0.28	1.19
Lgals3	0.7998	< 0.001	0.78	19.90
Cd68	0.7278	< 0.01	0.58	5.71
Fcgr1	0.9459	< 0.001	0.09	6.00
Tnfa	0.1963	< 0.001	0.14	0.45
Ccl2	0.9328	< 0.01	0.08	3.60
Mrc1	0.1436	< 0.05	0.47	0.81
Mrc2	< 0.05	< 0.05	0.29	-0.31
Clec10a	0.8919	< 0.01	0.11	2.26
Spp1	0.9950	<0.05	0.02	7.99
